# Which older people decline participation in a primary care trial of physical activity and why: insights from a mixed methods approach

**DOI:** 10.1186/1471-2318-14-46

**Published:** 2014-04-12

**Authors:** Annabelle Rogers, Tess Harris, Christina Victor, Alison Woodcock, Elizabeth Limb, Sally Kerry, Steve Iliffe, Peter Whincup, Ulf Ekelund, Carole Beighton, Michael Ussher, Fredrika Adams, Derek G Cook

**Affiliations:** 1 Population Health Research Institute, St George’s University of London, London SW17 ORE, UK; 2 Gerontology and Health Services Research Unit, Brunel University, London UB8 3PH, UK; 3 Psychology Department, Royal Holloway, University of London, London TW20 OEX, UK; 4 Pragmatic Clinical Trials Unit, Queen Mary’s University of London, London E12AT, UK; 5 Department of Population Health Sciences, University College, London NW3 2PF, UK; 6 MRC Epidemiology Unit, University of Cambridge, Cambridge CB2 OQQ, UK; 7 Department of Sport Medicine, Norwegian School of Sport Sciences, PO Box 4014, 0806, Oslo, Norway; 8 Faculty of Health and Social Care, London South Bank University, London SE1 0AA, UK

**Keywords:** Physical activity, Non-participation, Primary care, Older people, Recruitment

## Abstract

**Background:**

Physical activity is of vital importance to older peoples’ health. Physical activity intervention studies with older people often have low recruitment, yet little is known about non-participants.

**Methods:**

Patients aged 60–74 years from three UK general practices were invited to participate in a nurse-supported pedometer-based walking intervention. Demographic characteristics of 298 participants and 690 non-participants were compared. Health status and physical activity of 298 participants and 183 non-participants who completed a survey were compared using age, sex adjusted odds ratios (OR) (95% confidence intervals). 15 non-participants were interviewed to explore perceived barriers to participation.

**Results:**

Recruitment was 30% (298/988). Participants were more likely than non-participants to be female (54% v 47%; p = 0.04) and to live in affluent postcodes (73% v 62% in top quintile; p < 0.001). Participants were more likely than non-participants who completed the survey to have an occupational pension OR 2.06 (1.35-3.13), a limiting longstanding illness OR 1.72 (1.05-2.79) and less likely to report being active OR 0.55 (0.33-0.93) or walking fast OR 0.56 (0.37-0.84). Interviewees supported general practice-based physical activity studies, particularly walking, but barriers to participation included: already sufficiently active, reluctance to walk alone or at night, physical symptoms, depression, time constraints, trial equipment and duration.

**Conclusion:**

Gender and deprivation differences suggest some selection bias. However, trial participants reported more health problems and lower activity than non-participants who completed the survey, suggesting appropriate trial selection in a general practice population. Non-participant interviewees indicated that shorter interventions, addressing physical symptoms and promoting confidence in pursuing physical activity, might increase trial recruitment and uptake of practice-based physical activity endeavours.

## Background

Physical activity (PA) reduces the risk of over 20 adverse health conditions in older people, as well as improving emotional wellbeing [1]. Physical inactivity is the fourth leading cause of death worldwide [2] and a major cost burden on health services [1].

Primary care has a key role in encouraging older people to become more active [3] and primary care nurses often deliver new National Health Service (NHS) Health Checks, assessing PA levels using the General Practice Physical Activity Questionnaire (GPPAQ) [4] and advising on increasing PA in adults up to age 74 [5].

Primary care PA studies report low response rates (46% for questionnaires [6]; 6-35% for intervention studies [7,8]). Low recruitment can lead to selection biases, thereby threatening generalisation of results. However, evidence on selection bias is contradictory: some studies reporting that trial participants are more active [9,10], and have better health [11] and functional status than non-participants [7], with other studies reporting that they have poorer health [9,12]. Such contradictions hinder the translation of findings and highlight the importance of studying trial non-participants, including a qualitative component to understand better their decision.

The PACE-Lift trial is a three month intervention designed to increase walking, using pedometers, accelerometers and one-to-one nurse consultations for older primary care patients. The target number of patients to be recruited was 300. The protocol fully describes the study design and trial interventions [13].

### Aims

We aimed to elucidate factors influencing participation in a primary care PA trial in older adults by: 1) comparing demographic details of all those invited to participate; 2) comparing self-reported socio-demographic characteristics, health status and PA levels of participants with those of non-participants who completed a survey; and 3) exploring in interviews a sample of non-participants’ perceived barriers to participation.

## Methods

### Design

A two-phase mixed-methods sequential explanatory design was used. Phase one involved collection of quantitative data using general practice records and a questionnaire survey. Phase two involved semi-structured interviews with a sample of trial non-participants. The rationale for this approach was to explain quantitative results by exploring non-participants’ views in more breadth and depth [14].

### Setting

Three general practices in Oxfordshire and Berkshire, United Kingdom. Practices were selected that had the following: a list size >10,000 patients or >1400 patients aged 60–74 years; a practice nurse interested in carrying out the physical activity interventions; and the availability of a room for the research assistant.

### Subjects

**Figure 1:**
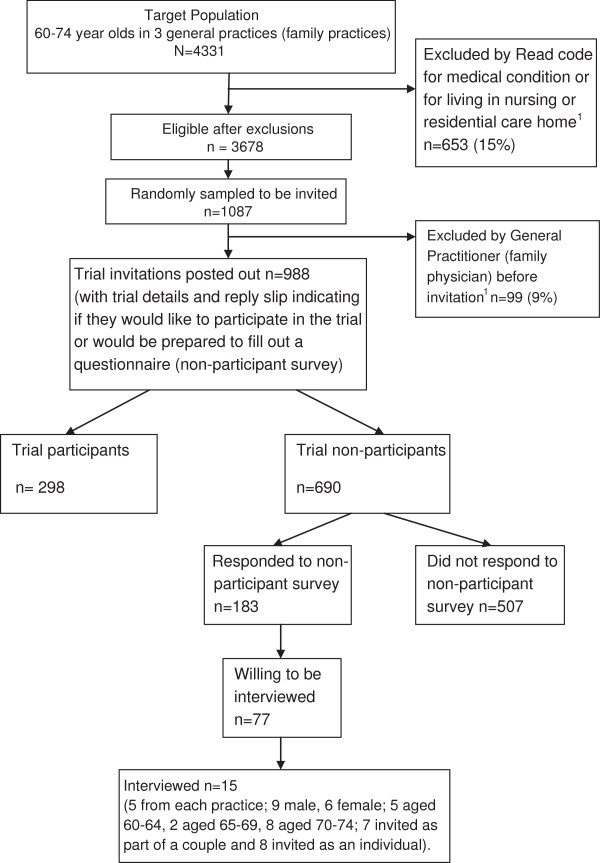
PACE-Lift Trial Recruitment and Selection of Non-Participant Interviewee Sample.

Patients aged 60–74 years registered at participating practices were invited to take part in the trial if they could walk outside and had no contraindications to increasing PA. Computerised primary care records were screened by Read codes for exclusions and random samples of households were selected. (If there were 2 members of a couple living at the same address who were both potentially eligible this was a ‘double’ household; if there was only one person potentially eligible this was a ‘single’ household). General practitioners (family physicians) then scrutinised these for further exclusions, before posting study invitations (Figure 1). The invitation included trial information and a response sheet with the options of i) trial participation, ii) completing a survey or iii) no further contact. There were 298 trial participants and 690 non participants, of whom 183 completed a questionnaire.

### Ethical approval

This was provided by NRES Committee Oxford C 11/H0606/2.

### Measures

#### Quantitative (Phase 1)

Postcode information recorded in computerised primary care registers allowed us to assign an index of multiple deprivation (IMD) score based on material deprivation measures, to rank individuals using national quintiles [15].

Participants and 183 non-participants completed a ‘Health and Physical Activity Baseline Survey’, (Table 1). Self-reported PA was assessed by GPPAQ [4].

**Table 1:** Comparisons between participants and all non-participants

	**Participants**	**All non-participants**	**Crude OR participating**	**Test for trend**
	**N = 298 n (%)**	**N = 690 n (%)**	**(95% CI)**	**p value**
**Age at posting invitation**				
60-64 years	113 (37.9)	254 (36.8)	1.0	0.38
65-69 years	104 (34.9)	222 (32.2)	1.05 (0.76, 1.45)	
70-75 years	81 (27.2)	214 (31.0)	0.85 (0.61, 1.19)	
**Gender**				
Female	160 (53.7)	322 (46.7)	1.0	0.04
Male	138 (46.3)	368 (53.3)	0.75 (0.57, 0.99)	
**Invited as**				
Couple	149 (50.0)	322 (46.7)	1.0	0.34
Individual	149 (50.0)	368 (53.3)	0.88 (0.67, 1.15)	
**National quintiles of Index of Multiple Deprivation Rank**^**1**^				
1 (most deprived)	2 (0.7)	12 (1.7)	0.32 (0.07, 1.46)	<0.001
2)	9 (3.0)	32 (4.6)	0.55 (0.26, 1.17)	
3	18 (6.0)	81 (11.7)	0.43 (0.25,0.74)	
4	51 (17.1)	140 (20.3)	0.71 (0.50. 1.02)	
5 (least deprived)	218 (73.2)	425 (61.6)	1.0	

^1^Index of Multiple Deprivation Rank from national quintiles [15].

The odds ratios (ORs) for participating by various characteristics were estimated using logistic regression, adjusting as appropriate for possible confounding variables. Trends in ORs across IMD fifths were modelled by fitting IMD fifths as a continuous categorical variable coded 1–5. Trends across GPPAQ and General Health categories were similarly assessed, while a likelihood ratio test was used to test for variability in ORs between marital status categories.

#### Qualitative (Phase 2)

Permission to contact trial non-participants for a 10-minute interview was requested on the survey. Of the 183 non-participants surveyed, 77 who gave permission and 106 who did not were compared in terms of age, gender, whether invited as a couple or not, BMI and self-reported PA (GPPAQ). Fifteen of the 77 were telephoned and interviewed (Figure 1). Interviewees were purposively selected to provide: males and females of varying ages (60–74), invited to participate as an individual or a couple, samples from all three practices. Face-to-face or telephone interviews were offered and written or audio-recorded consent obtained. Recruitment stopped when no new themes were identified.

### Development of interview schedule

The interviewer (AR) was a research assistant unknown to the interviewees. The semi-structured interview schedule was developed collaboratively and iteratively between AR, CV, AW and TH.

All interviews were initiated with an open question: *What was your main reason for deciding not to take part in the PACE-Lift study?* Probing then used previous questionnaire responses and additional questions. Possible reasons explored were: lack of time; unable/uninterested to increase PA; already physically active; not interested in research; do not want to be allocated by chance; length of programme; travel difficulties; individual versus group consultations; and trial equipment. Further trial issues were then explored: venue (e.g. general practice versus leisure centre); walking versus other PA interventions; group versus individual nurse contact; other factors that would have encouraged involvement.

### Qualitative analysis

The interviews were transcribed verbatim. Transcripts were subjected to an initial analysis stage ‘data reduction’ [16] involving selecting and transforming the data as meanings and insights from the words of interviewees. AR uploaded the data onto QSR Nvivo (V9.0, Scientific Software) and read each transcript several times. This enabled identification of patterns both within and across transcripts with the aim of ensuring that recurrent codes were subject to closer scrutiny; alternative interpretations were explored, as advised in the qualitative analysis coding manual [16]. The initial thematic framework was discussed during monthly face-to-face meetings with co-researchers who had read and coded the 15 transcripts independently, discrepancies were resolved and additional codes refined until consensus was reached. Nvivo keyword searches were used to identify overlooked material.

## Results

### Comparison of participants and all non-participants using data from computerised primary care records (Table 1)

Trial recruitment was 30% (298/988). Participants were slightly but not significantly younger than non-participants (Table 1.) They were more likely to be female (p = 0.04) but being invited as a couple did not affect response. More participants lived in the most affluent quintile of Index of Multiple Deprivation, 73%, compared with 62% of non-participants (p < 0.001). However, the overall sample from the three practices was relatively affluent, with 65% (643/988) living in the least deprived quintile of deprivation, compared to 20% nationally [15].

### Comparison of participants and non-participants completing the survey (Table 2)

**Table 2:** Comparisons between participants and non-participants who responded to the survey

		**Non-participants completing survey**	**Crude OR for participating**	**Adj OR for participating**	**Test for trend**
	**Participants**				
	**N = 298 n (%)**	**N = 183 n (%)**	**(95% CI)**	**(Age & sex adj)**	**p value**
**Age at posting invitation**					0.07
60-64	113 (37.9)	59 (32.2)	1.0		
65-69	104 (34.9)	59 (32.2)	0.92 (0.59, 1.44)		
70-75	81 (27.2)	65 (35.5)	0.65 (0.41, 1.02)		
**Gender**					
Female	160 (53.7)	322 (46.7)	1.0		0.47
Male	138 (46.3)	91 (49.7)	0.87 (0.60, 1.26)		
**Invited as**					0.76
Couple	149 (50.0)	94 (51.4)	1.00	1.00	
Individual	149 (50.0)	89 (48.6)	1.06 (0.73, 1.53)	1.06 (0.73, 1.54)	
**National quintiles of IMD Rank**^**1**^					0.25
1 (most deprived)	2 (0.7)	2 (1.1)	0.61 (0.08, 4.35)	0.70 (0.10, 5.11)	
2	9 (3.0)	10 (5.5)	0.54 (0.22, 1.38)	0.55 (0.22, 1.39)	
3	18 (6.0)	15 (8.2)	0.73 (0.35, 1.49)	0.69 (0.34, 1.43)	
4	51 (17.1)	24 (13.1)	1.29 (0.76, 2.19)	1.28 (0.75, 2.18)	
5 (least deprived)	218 (73.2)	132 (72.1)	1.00	1.00	
**Marital status**					0.58^8^
Married	240 (80.8)	149 (82.8)	1.00	1.00	
Widowed	21 (7.1)	8 (4.4)	1.63 (0.70, 3.77)	1.78 (0.75, 4.25)	
Divorced or Separated	24 (8.1)	14 (7.8)	1.06 (0.53, 2.12)	1.02 (0.51, 2.04)	
Single	12 (4.0)	9 (5.0)	0.83 (0.34, 2.01)	0.86 (0.35, 2.10)	
**Retired**	193 (66.8)	119 (72.1)	0.78 (0.51, 1.18)	0.81 (0.52, 1.27)	0.36
**Occupational pension**	214 (75.6)	107 (61.9)	1.91 (1.27, 2.88)	2.06 (1.35, 3.13)	0.001
**White ethnicity**	290 (98.6)	181 (100.0)			
**Current smoker**	16 (5.5)	14 (8.0)	0.67 (0.32, 1.41)	0.66 (0.31, 1.39)	0.27
**Body Mass Index**^**2**^					0.10
Overweight or obese (≥25 kg/m^2^)	200 (67.1)	96 (59.6)	1.38 (0.93, 2.06)	1.40 (0.93, 2.09)	
**General health**					0.32
Very good or good	260 (88.7)	150 (85.2)	1.00	1.00	
Fair	30 (10.2)	24 (13.6)	0.72 (0.41, 1.28)	0.74 (0.41, 1.31)	
Bad or very bad	3 (1.0)	2 (1.1)	0.87 (0.14, 5.24)	0.82 (0.13, 5.00)	
**Limiting long-standing illness**	72 (25.4)	29 (16.8)	1.69 (1.05, 2.74)	1.72 (1.05, 2.79)	0.03
**One or more chronic disease**^**3**^	207 (69.5)	107 (58.5)	1.62 (1.10, 2.37)	1.68 (1.14, 2.47)	0.009
**Uses a walking aid**^**4**^	8 (2.7)	5 (2.9)	0.95 (0.31, 2.94)	0.98 (0.31, 3.07)	0.97
**Fallen in last year**^**4**^	48 (16.2)	38 (21.6)	0.70 (0.44, 1.12)	0.66 (0.41, 1.08)	0.10
**≥ 4 medications a day**	60 (20.4)	40 (23.0)	0.86 (0.55, 1.35)	0.90 (0.56, 1.42)	0.64
**EQ-5D**^**5**^					
Mobility: some problems	37 (12.6)	29 (16.4)	0.73 (0.43, 1.24)	0.74 (0.43, 1.25)	0.26
Self-care: some problems	4 (1.4)	2 (1.1)	1.18 (0.21, 6.51)	1.06 (0.19, 5.88)	0.95
Usual activities: some problems	38 (12.8)	26 (14.7)	0.85 (0.50, 1.46)	0.85 (0.49, 1.46)	0.55
Pain/discomfort: some pain	120 (41.2)	58 (33.1)	1.42 (0.96, 2.09)	1.43 (0.96, 2.12)	0.08
Anxiety/Depression: yes	34 (11.5)	23 (13.2)	0.85 (0.48, 1.50)	0.85 (0.48, 1.51)	0.58
**General practice physical activity**					
**Questionnaire (GPPAQ)**^**6**^					
Inactive	152 (52.6)	72 (52.6)	1.00	1.00	0.03
Moderately inactive	35 (12.1)	22 (12.1)	0.75 (0.41, 1.38)	0.71 (0.39, 1.31)	
Moderately active	55 (19.0)	32 (19.0)	0.81 (0.48, 1.37)	0.78 (0.46, 1.33)	
Active	47 (16.3)	39 (16.3)	0.57 (0.34, 0.95)	0.55 (0.33, 0.93)	
**Brisk or fast walking pace**^**6**^	87 (30.3)	68 (41.5)	0.61 (0.41, 0.92)	0.56 (0.37, 0.84)	0.006
**Reasons for non-participation**^**7**^					
Already physically active		101 (67.3)			
Not enough time		66 (44.0)			
Not interested to increase PA		37 (24.7)			
Not able to increase physical activity		32 (21.3)			
Not wanting to be allocated by chance		20 (13.3)			
Not interested in research		12 (8.0)			

^1^Index of Multiple Deprivation Rank from national quintiles [15].

^2^Body mass index = weight(kg)/height(m)^2^ based on measured values for participants and self-reported height and weight for non-participants, height and weight were not available for 22 (13%) non-participants.

^3^Chronic disease score adapted from self-report checklist of doctor diagnosed chronic medical conditions used in Alameda County Study [17].

^4^Use of walking aid and number of falls in previous year.

^5^EQ-5D health outcome measures [18].

^6^Self-reported physical activity over last 7 days (General Practice Physical Activity Questionnaire; GPPAQ) [4].

^7^Reasons for non-participation, 6 possible reasons for non-participation in trial, not mutually exclusive (response options yes/no/unsure).

^8^Test for heterogeneity between groups.

Trial participants and non-participants completing the survey did not differ in marital or retirement status or whether they had been invited to take part as a couple. Smoking status and ethnicity showed no differences, but as only 6% were smokers and only about 1% were non-white, these comparisons lacked statistical power. However, participants were more likely to have an occupational pension, adjusted OR 2.06 (1.35-3.13). Trial participation was positively associated with having a limiting long-standing illness, adjusted OR 1.72 (1.05-2.79) and having one or more chronic diseases, adjusted OR 1.68 (1.14-2.47). Participants tended to be overweight or obese adjusted OR 1.40 (0.93-2.09) and in pain adjusted OR 1.43 (0.96-2.12), but neither difference was statistically significant. General health, use of a walking aid, falls, number of medications, mobility or self-care problems, anxiety or depression, showed no association with participation. Participation was significantly associated with lower self-reported PA: slower walking pace, adjusted OR 0.56 (0.37-0.84) and lower activity levels, adjusted OR 0.55 (0.33-0.93). The main reasons given on the survey for non-participation were: already physically active (67%); time constraints (44%); lack of interest (25%).

### Qualitative interview participant selection and findings (Table 3)

**Table 3:** Key themes from the transcripts identifying interviewees’ reasoning behind their decision not to participate in the trial

Themes		
Trial design and delivery	Physical-environmental:	• Climate/seasonal factors “It’s a bit off-putting I think really, as it’s very wet and gets dark early -you don’t want to be walking in it”
		• GP practice venue was generally supported “At the practice, it’s easier, don’t change that” “I walk down there anyway when I go and see the doctor”
		• Use of trial equipment specifically the accelerometer was not supported due to discomfort “It would put me off a bit because I’m waiting to have a heart monitor - I can’t cope with two” and inadequate recording “The monitor only indicates steps and I have problems with my feet so I ride a bike and that doesn’t record so well on the monitor”
	Focus on walking:	• The trial’s focus on walking emerged as a preferred physical activity of older people and was favoured over other forms of physical activity “Walking is fine. It’s probably the best thing you can do at our age”
	Socio-cultural influences:	• Individual versus group consultation was generally preferred “I’m not very good in groups I would say. I think I would prefer one-to-one”
		• Social support was deemed important from peers, spouse or family “I discussed it (the trial) with my children and they said it was a good idea”
	Altruistic factors:	• Older people are supportive of medical research and would like to help if they could “I would like to think of a way to help them in any way, research or whatever”
Current health and activity	Personal competence:	• Many believed they were already doing enough physical activity “I don’t really think it was a necessary thing for me to do because I think I do a lot of exercise anyway
		• Real and perceived medical problems and fear of such problems were significant barriers to regular physical activity “I think that was one of my main worries, that I just felt my knee would get worse”
		• For some, psychological barriers such as lack of confidence and depression posed significant challenges to participation “I suffer a lot of pain all the time, 24 hours a day, and it gets me depressed”
Practical reasoning	Time-related factors:	• Some devoted a lot of time to other commitments: work, caring for one’s family, recreation “I’m just really rather busy, I play a lot of sport and look after various people and I just don’t feel I would have the time to fit it all in”
		• The main 3 month involvement in the trial was thought to be too long “I think 3 months is a bit of a long time”
	Behavioural attributes:	• Changing behaviour appeared problematic due to existing routines "I've got into a routine I like to stay in. If I go and do anything different, it sounds silly, but I get a bit uptight"
		• Many believed they did not need help and could increase physical activity on their own “Exercise is in my own hands if I choose to do something about it as opposed to being part of an organised team to do it”
		• Lack of interest in physical activity: reluctance to do activities that are not enjoyed “I don’t want to spend every minute I’ve got free thinking I’ve got to do exercise”
		• Acknowledgement of ‘slowing down’ in retirement "I find an awful lot of people our age, they think about exercise but very few do anything about it. As they get older, they slow down and can’t be bothered”

Amongst trial non-participants, the 77 who agreed to be approached for an interview were compared to those who did not (106). There were no statistically significant differences in age group 32% (24/77) versus 39% (41/106) age 70–75 (p = 0.37); gender 53% (41/77) versus 47% (50/106) male (p = 0.42); invited as a couple 51% (39/77) versus 52% (55/106) (p = 0.87); BMI 54% (37/69) versus 64% (59/92) overweight or obese (p = 0.18) or IMD score 71% (55/77) versus 73% (77/106) in least deprived quintile (p = 0.59). There was a significant difference in self-reported PA, with those who agreed to be interviewed more likely to answer the question and to self-report higher PA levels, 53% (40/76) versus 35% (31/89) categorised as “active” on GPPAQ (p = 0.021). Table 3 summarises key themes from the 15 interviews conducted. Barriers to trial participation included reluctance to walk either alone, in the evening, or in bad weather, linked to a fear of falling in wet weather and reduced confidence in pursuing PA. Poor physical and psychological health, symptoms of medical conditions, particularly arthritis and joint pain, psychological health concerns such as depression and low energy levels reduced the desire to exercise, as did a fear of “overdoing things”, with the view that PA might cause “more harm than good”. The predominant explanation for non-participation was the perception that they were already doing enough PA; they were aware of the health benefits of keeping active and believed that the trial would provide minimal benefit. The commitment required to participate was mentioned by those who expressed the desire to take on fewer commitments, those reluctant to change existing routines and where interviewee’s priorities (work, family and recreation) were given priority over pursuit of PA. Some expressed concerns about lengthy trial duration and uncomfortable trial equipment.

In terms of positive trial features, interviewees supported the focus on walking over other PA for their age group and the involvement of general practice (family practice).

## Discussion

### Main findings

The study achieved a good recruitment rate for a primary care PA trial [7,8,19,20], in a relatively affluent white population. Within this context, participants were more likely to come from affluent postcodes and be female, but being invited as an individual or as part of a couple did not affect response. Non-participants completing a survey were less likely than participants to report health problems and more likely to report being physically active, suggesting appropriate trial self-selection. This suggests that primary care recruitment could encourage those with more illnesses and primary care contact to join PA trials and fits with recommendations to improve recruitment of older people into research, particularly clinical trials [21]. The qualitative results supported the survey finding that some non-participants thought they were already sufficiently active, whilst physical symptoms, depression, time restraints, trial equipment and duration were important barriers to participation for others.

### Study strengths and limitations

The main strength was the mixed methods approach of the study, with the ability to examine routinely collected demographic data on all those invited to participate, supported by more detailed data on health and PA levels of non-participants completing a survey and interview data from a purposively selected sample. It is difficult to obtain more than demographic information on non-participants [6,9,10], so any information is valuable from a public health perspective for exploring reasons why older people do not want to participate in PA interventions. Approximately 50% of patients either participated in the trial or the survey, confirming the value of this work. However, we cannot assume that reasons given by survey responders or by the small group of interviewees, apply to all trial non-participants; indeed whilst those agreeing to be interviewed were similar on most measures to those not agreeing, they reported higher PA levels. We were limited in the socio-economic measures assessed: only postcode and therefore IMD score [15] for all non-participants; only receipt of an occupational pension for those non-participants responding to the questionnaire. A further limitation was that the BMI of non-participants was calculated from self-reported height and weight, whereas these were objectively measured in participants. This could have led to under-reporting of BMI in non-participants, inflating the difference between participants and non-participants.

### Comparison with other studies

Our recruitment rate of 30% compares well to other primary care physical activity interventions in this age group: 6% [7], 14% [20], 17% [19], 35% [8], reflecting the relatively affluent setting. Participants were more likely than non-participants to report having a limiting longstanding illness, and one or more chronic diseases (and a tendency, although not statistically significant, to suffer pain and to be overweight or obese) supporting work which found that those with a greater disease history and primary care contact were more likely to participate in primary care PA studies [9,12] and refuting a ‘healthy volunteer effect’ [11]. Participants reported being less active and having lower walking speeds than the non-participants surveyed, consistent with their poorer physical health. This contrasts with our previous work where participants reported more PA [9]. However, that was an observational study, objectively measuring usual PA, and may have attracted those interested in PA. The current study required commitment to increase PA levels over 3–12 months [13], so those who felt they were sufficiently active may have been less likely to participate.

The interviews provided insight into perceived barriers to PA trial participation. Already considering themselves sufficiently active was a common reason for non-participation, though this may be due to higher self-reported PA levels in those who agreed to be interviewed and an overestimation of what they actually did [6,22]. However, several interviewees confirmed that they were not physically active. Despite reporting an understanding of health benefits, their health concerns impeded them from exercising. Current poor health, including joint pain, as well as psychological factors such as depression, fear of falling and low energy were mentioned as reasons for not participating in PA, consistent with other work [6]. These findings have implications for primary care-based trials, suggesting that patients need tailored information concerning symptom management, highlighting safety and joint protection and evidence that PA can be beneficial and may alleviate painful joints [23]. Furthermore, there is a need to emphasise mental health benefits, so that a perceived barrier to exercise may become a potential benefit [24].

In common with previous studies [25,26], lack of time was a common reason for non-participation. This was linked to work and recreational commitments, in addition to ‘slowing down’ in retirement, taking things at a leisurely pace. Although interviewees acknowledged that no programme could accommodate everyone, juggling competing responsibilities such as work and family was a barrier. Similar to previous findings [27], some interviewees cited long working hours and related fatigue as reasons for finding exercise difficult to fit in. The majority talked about the importance of maintaining a balance between work and leisure, and the possible disruption caused by increased PA. A change in emphasis is needed to ensure that older people understand the importance of exercising, and recognise that exercise can be pleasurable. These qualitative findings illustrate the importance of tailoring PA to match individual interests when designing interventions [28]. Whilst this was an important feature of our intervention, it could have been emphasised more in recruitment materials, pre-empting potential barriers.

A reluctance to go out in the evening and to go out alone prevented some from participating, as highlighted previously [6]. Trial invitations were staggered over a year, and those approached over winter were discouraged by seasonal factors and fear of falling. This highlights the importance of safety and confidence in PA and the need to address self-efficacy, which is positively related to PA engagement by older adults [29]. Organised group exercise within PA interventions may enable participants to meet others of a similar fitness level and may reduce safety concerns [30]. However, interviewees in our study supported walking as the best choice of activity for this age group and primary care as an appropriate venue for the intervention, with practice nurse support, and this model has been successful previously [31]. Outdoor walking interventions in primary care could usefully recruit in the spring or summer, building new habits before the weather deteriorates.

## Conclusions

This mixed-method approach enabled a greater understanding of factors influencing older adults’ participation in PA studies. The quantitative comparisons showed some selection bias into the trial, with participants more likely to be female and living in less deprived areas. However, participants had more health problems and lower activity levels than non-participants who completed the survey, suggesting appropriate trial selection in a general practice population. Interviews with a small sample of non-participants revealed complex interacting physical, psychological and environmental factors underpinning the decision against trial participation. PA promotion needs to raise awareness of both physical and mental health benefits, whilst addressing physical symptoms and lack of confidence. To improve trial participation and uptake of practice-based PA endeavours, the needs of older adults should be addressed at recruitment.

## Abbreviations

PA: Physical activity; NHS: National Health Service; GPPAQ: General Practice Physical Activity Questionnaire; OR: Odds Ratio; CI: Confidence Interval.

## Competing interests

The authors declare that they have no competing interests.

## Authors’ contributions

TH, DC and CV conceived the idea for the PACE-Lift study. TH, DC, CV, SK, AW, SI, UE, PW, CB, MU and FA participated in the design of the study, including the idea of a comparison of participants and non-participants. CV, AW, TH and AR designed the qualitative aspects of the study. AR carried out all the non-participant interviews and qualitative analyses. EL carried out the statistical comparisons between participants and non-participants. All the authors have read and approved the final manuscript.

## Pre-publication history

The pre-publication history for this paper can be accessed here:

http://www.biomedcentral.com/1471-2318/14/46/prepub

## References

[B1] Department of Health Start Active, Stay Active: A report on physical activity for health from the four home countries' Chief Medical Officers 2011

[B2] Hallal PC Andersen LB Bull FC Guthold R Haskell W Ekelund U Global physical activity levels: surveillance progress, pitfalls, and prospects Lancet 2012 14 247 257 10.1016/S0140-6736(12)60646-1 22818937

[B3] National Institute for Health and Clinical Excellence Walking and cycling. Local measures to promote walking and cycling as forms of travel or recreation 2012 London: NICE Public Health Guidance 41

[B4] Physical Activity Policy HIDDoH The General Practice Physical Activity Questionnaire (GPPAQ) 2009 London: Department of Health

[B5] NHS Health Checks Programme Putting prevention first: NHS Health Checks: Vascular Risk Assessment and Management Best Practice Guidelines 2009

[B6] Crombie IK Irvine L Williams B McGinnis AR Slane PW Alder EM McMurdo ME Why older people do not participate in leisure time physical activity: a survey of activity levels, beliefs and deterrents Age Ageing 2004 14 287 292 10.1093/ageing/afh089 15082435

[B7] Tully MA Cupples ME Chan WS McGlade K Young IS Brisk walking, fitness, and cardiovascular risk: a randomized controlled trial in primary care Prev Med 2005 14 622 628 10.1016/j.ypmed.2004.11.030 15917061

[B8] Stevens W Hillsdon M Thorogood M McArdle D Cost-effectiveness of a primary care based physical activity intervention in 45–74 year old men and women: a randomised controlled trial Br J Sports Med 1998 14 236 241 10.1136/bjsm.32.3.236 9773174 PMC1756094

[B9] Harris TJ Victor CR Carey IM Adams R Cook DG Less healthy, but more active: opposing selection biases when recruiting older people to a physical activity study through primary care BMC Public Health 2008 14 182 10.1186/1471-2458-8-182 18505574 PMC2426698

[B10] Golomb BA Chan VT Evans MA Koperski S White HL Criqui MH The older the better: are elderly study participants more non-representative? A cross-sectional analysis of clinical trial and observational study samples BMJ Open 2012 14 e000833 doi:10.1136/bmjopen-2012-000833 23242479 10.1136/bmjopen-2012-000833 PMC3533104

[B11] van Heuvelen MJ Hochstenbach JB Brouwer WH de Greef MH Zijlstra GA van Jaarsveld E Kempen GI van Sonderen E Ormel J Mulder T Differences between participants and non-participants in an RCT on physical activity and psychological interventions for older persons Aging Clin Exp Res 2005 14 236 245 10.1007/BF03324603 16110738

[B12] Ives DG Traven ND Kuller LH Schulz R Selection bias and nonresponse to health promotion in older adults Epidemiology 1994 14 456 461 10.1097/00001648-199407000-00013 7918817

[B13] Harris T Kerry S Victor CR Ekelund U Woodcock A Iliffe S Whincup PH Beighton C Ussher M David L Brewin D Adams R Rogers A Cook DG Randomised controlled trial of a complex intervention by primary care nurses to increase walking in patients aged 60–74 years: protocol of the PACE-Lift (Pedometer Accelerometer Consultation Evaluation - Lift) trial BMC Public Health 2013 14 5 10.1186/1471-2458-13-5 23289648 PMC3543841

[B14] Creswell JW Plano Clark VL Designing and conducting mixed methods research 2011 London: Sage

[B15] Noble M McLennan D Wilkinson K Whitworth A Barnes H The English Indices of Deprivation 2007 2008 London: Department of Communities and Local Government

[B16] Miles M Huberman M Qualitative Data Analysis: An expanded source book 1994 Thousand Oaks CA: Sage Publications

[B17] Roberts RE Kaplan GA Shema SJ Strawbridge WJ Prevalence and correlates of depression in an aging cohort: the Alameda County Study J Gerontol B Psychol Sci Soc Sci 1997 14 S252 S258 9310097 10.1093/geronb/52b.5.s252

[B18] Brooks R EuroQol: the current state of play Health Policy 1996 14 53 72 10.1016/0168-8510(96)00822-6 10158943

[B19] Little P Dorward M Gralton S Hammerton L Pillinger J White P Moore M McKenna J Payne S A randomised controlled trial of three pragmatic approaches to initiate increased physical activity in sedentary patients with risk factors for cardiovascular disease Br J Gen Pract 2004 14 189 195 15006124 PMC1314829

[B20] Sugden JA Sniehotta FF Donnan PT Boyle P Johnston DW McMurdo ME The feasibility of using pedometers and brief advice to increase activity in sedentary older women–a pilot study BMC Health Serv Res 2008 14 169 doi:10.1186/1472-6963-8-169 10.1186/1472-6963-8-169 18691392 PMC2527003

[B21] McMurdo ME Roberts H Parker S Wyatt N May H Goodman C Jackson S Gladman J O'Mahoney S Ali K Dickinson E Edison P Dyer C Improving recruitment of older people to research through good practice Age Ageing 2011 14 659 665 10.1093/ageing/afr115 21911335

[B22] Joint Health Surveys Unit Health Survey for England 2008 Physical activity & fitness 2009 The NHS Information Centre for health & social care

[B23] Penninx BW Messier SP Rejeski WJ Williamson JD DiBari M Cavazzini C Applegate WB Pahor M Physical exercise and the prevention of disability in activities of daily living in older persons with osteoarthritis Arch Intern Med 2001 14 2309 2316 10.1001/archinte.161.19.2309 11606146

[B24] Camacho TC Roberts RE Lazarus NB Kaplan GA Cohen RD Physical activity and depression: evidence from the Alameda County Study Am J Epidemiol 1991 14 220 231 1862805 10.1093/oxfordjournals.aje.a116074

[B25] Chao D Foy CG Farmer D Exercise adherence among older adults: challenges and strategies Control Clin Trials 2000 14 212S 217S 10.1016/S0197-2456(00)00081-7 11018578

[B26] Cohen-Mansfield J Marx MS Guralnik JM Motivators and barriers to exercise in an older community dwelling population J Ageing Phys Act 2003 14 242 253

[B27] Costello E Kafchinski M Vrazel J Sullivan P Motivators, barriers, and beliefs regarding physical activity in an older adult population J Geriatr Phys Ther 2011 14 138 147 10.1519/JPT.0b013e31820e0e71 21937904

[B28] Brawley LR Rejeski WJ King AC Promoting physical activity for older adults: the challenges for changing behavior Am J Prev Med 2003 14 172 183 10.1016/S0749-3797(03)00182-X 14552942

[B29] Brassington GS Atienza AA Perczek RE DiLorenzo TM King AC Intervention-related cognitive versus social mediators of exercise adherence in the elderly Am J Prev Med 2002 14 80 86 10.1016/S0749-3797(02)00477-4 12133741

[B30] Iliffe S Kendrick D Morris R Skelton D Gage H Dinan S Stevens Z Pearl M Masud T Multi-centre cluster randomised trial comparing a community group exercise programme with home based exercise with usual care for people aged 65 and over in primary care: protocol of the ProAct 65+ trial Trials 2010 14 6 10.1186/1745-6215-11-6 20082696 PMC2821309

[B31] Lawton BA Rose SB Elley CR Dowell AC Fenton A Moyes SA Exercise on prescription for women aged 40–74 recruited through primary care: two year randomised controlled trial BMJ 2008 14 a2509 10.1136/bmj.a2509 19074218 PMC2769033

